# The Effects of Various Amendments on Trace Element Stabilization in Acidic, Neutral, and Alkali Soil with Similar Pollution Index

**DOI:** 10.1371/journal.pone.0166335

**Published:** 2016-11-11

**Authors:** Min-Suk Kim, Hyun-Gi Min, Sang-Hwan Lee, Jeong-Gyu Kim

**Affiliations:** 1 O-Jeong Eco-Resilience Institute, Korea University, Seoul, Republic of Korea; 2 Division of Environmental Science and Ecological Engineering, College of Life and Environmental Sciences, Korea University, Seoul, Republic of Korea; 3 Soil Remediation Team, Mine Reclamation Corporation, Wonju, Republic of Korea; University of Vigo, SPAIN

## Abstract

Many studies have examined the application of soil amendments, including pH change-induced immobilizers, adsorbents, and organic materials, for soil remediation. This study evaluated the effects of various amendments on trace element stabilization and phytotoxicity, depending on the initial soil pH in acid, neutral, and alkali conditions. As in all types of soils, Fe and Ca were well stabilized on adsorption sites. There was an effect from pH control or adsorption mechanisms on the stabilization of cationic trace elements from inorganic amendments in acidic and neutral soil. Furthermore, acid mine drainage sludge has shown great potential for stabilizing most trace elements. In a phytotoxicity test, the ratio of the bioavailable fraction to the pseudo-total fraction significantly affected the uptake of trace elements by bok choy. While inorganic amendments efficiently decreased the bioavailability of trace elements, significant effects from organic amendments were not noticeable due to the short-term cultivation period. Therefore, the application of organic amendments for stabilizing trace elements in agricultural soil requires further study.

## Introduction

Soil pollution from trace elements is one of the major environmental issues across the world [1]. Recently, among the many remediation technologies used on contaminated soils, chemical stabilization has been widely studied because of its efficiency, economic feasibility, and ability to prevent secondary pollution [2] The goal of stabilization is to reduce the harmfulness of trace elements by decreasing their mobility or bioavailability in the soil environment, but not changing their total concentration [3].

Many studies have examined the successful stabilization of trace elements in the soil using various soil amendments, including pH change-induced immobilizers, inorganic adsorbents, and organic materials [3]. Several conventional amendments (e.g., sludge, organic matter, limestone, etc.) have been widely used for trace element stabilization in soil, and extensive research of the use of industrial byproducts has also been recently examined [1, 3, 4]. Yang and Kang [5] found red mud and bio-solid stabilization effects on trace elements, and Lee et al. [1] demonstrated that bone meal and bottom ash could reduce the extractability and bioavailability of soil trace elements.

Chemical stabilization involves several mechanisms. Most pH change-inducing amendments increased the soil pH, resulting in the reduced mobility of cationic trace elements; furthermore, many adsorption-based amendments adsorbed not only cationic trace elements but also anionic metalloids [3]. Organic matter has also been used to provide adsorption sites and improve soil quality [6].

However, these various chemical stabilization mechanisms among trace elements did not work independently in the soil environment and were affected by several soil properties, especially the soil pH [7,8]. Chuan et al. [8] found that the sorption and solubility of metal on Fe-Mn oxyhydroxide sites were highly dependent on the soil pH. Smith [9] demonstrated that the pH of soil amended with sludge could be significantly affected by trace elements uptake by ryegrass. Furthermore, the availability of trace elements in the soil environment may be heavily affected by dissolved organic carbon (DOC), which is further affected by the soil pH and competition among ion species [3]. In this way, the soil pH influences trace element availability directly or indirectly. Nevertheless, most studies of soil amendments targeted only the stabilization efficiency; however, studies describing the effectiveness of soil pH on stabilization efficiency remain insufficient.

Therefore, in this study, we sought to evaluate the effects of various amendments on trace element stabilization and phytotoxicity at different pH levels in soil contaminated by trace elements at similar levels using both chemical and biological assessments.

## Materials and Methods

### Experimental set-up

We used the soils collected from private lands and the owner gave permission to conduct the study on these sites.

Three types of trace elements in contaminated soils were collected from different pH soils. Acidic soils (BH) were obtained from the Pungjeong mining area in Bonhwa-gun, Gyeongbuk Province, Korea. Neutral soils (GM) were obtained from the Gahak mining area in Gwangmyeong-si, Gyeonggi Province, Korea. Alkali soils (DY) were obtained from the Eugene mining area in Danyang-gun, Chungbuk Province, Korea. All of the soil sampling sites consisted of agricultural land adjacent to an abandoned mining area. The soil samples were air-dried and passed through a 2-mm sieve.

Six types of amendments were examined: steel slang (SS), waste lime (WL), acid mine drainage sludge (AMDS), bone meal (BM), pig manure (PM), and spent coffee ground char (SCGC). SS, WL, and AMDS were obtained from a steel plant and a soda ash plant and the sludge from an acid mine drainage treatment facility, respectively, at the Hamtae mine in Gangwon Province, Korea. BM and PM were obtained from a commercial fertilizer vendor. To produce SCGC, spent coffee grounds (SCG) were obtained from a local coffee shop in Seoul, Korea. The SCG were slowly pyrolyzed in a temperature-controlled electric furnace. The SCG were heated at approximately 20°C/min up to 400°C and were sustained there for 30 min. The final SCGC yield was 36%. All of the amendments were air-dried, ground, and sieved to less than 0.5 mm before storage in polyethylene bottles.

The pH and electrical conductivity (EC) of the soils and amendments were measured in a 1:5 suspension of solid:water using a combined pH-EC meter (Thermo Orion 920A); the weight loss-on-ignition was conducted to determine both the water and carbon content at 550°C over 24 hours [10]. Pseudo-total trace element (As, Cd, Cu, Pb, Zn) concentrations were determined by digesting the soils samples with concentrated HCl (9 mL) and HNO_3_ (3 mL) at 120°C and further analysis using commercially available temperature-controlled sand digestion technology. The digested suspension was filtered through Whatman No. 42 filter paper, and the trace elements concentrations in the filtered solution were determined using an inductively coupled plasma optical emission spectrometer (ICP-OES, 730 Series, Agilent, USA). The integrated pollution index (IPI) of each soil was calculated by the following from Wei and Yang [11]. The IPI was defined as the mean value of the pollution index (PI) of each trace element. In this study, the PI of each element was calculated as the ratio of the metal concentrations in the environment to the background concentration in Korea.

The amendments were applied to each soil at a 2% w/w ratio and were mixed thoroughly for homogeneity; the samples were equilibrated for 4 weeks while maintaining the soil moisture at approximately 60% of the soils’ water holding capacity (WHC) and were thoroughly mixed at weekly intervals. The WHC was measured by soaking the soil samples in water for 2 hours and then draining for 24 hours [12]. The effects of the amendments on the bioavailable trace element concentrations were evaluated using a Mehlich-3 solution [13] for chemical assessment [14–16].

### Phytotoxicity test for biological assessment

The short-term cultivation of bok choy (*Brassica campestris* L. ssp. chinensis Jusl.) was conducted for biological assessment. Twelve seeds were placed in 60 × 15 mm plastic Petri dishes containing 35 g of amendment-treated soils after 4 weeks of aging that were subsequently cultivated for 2 weeks. The Petri dishes were randomly placed in growth chambers under controlled conditions, and each treatment was repeated in triplicate. The moisture content was maintained at approximately 60% of the soil WHC; the light conditions included 16 hours of daylight and 8 hours of darkness per day; and the temperature was 24±2°C. Two weeks after sowing, the plants were harvested and washed with distilled water and the shoot elongation was immediately measured using an image analyzer program (WinRhizo 5.0a, Reagent, Canada) and a desktop scanner (Epson Perfection V700). The shoot elongation of bok choy was calculated for each seedling relative to the germination success rate (80%) in the control group and expressed in cm seedling^-1^. After determining the shoot elongation, the fresh weight of the bok choy shoots were also measured (mg seedling^-1^) and were then dried in a fan-forced oven at 60°C for 48 hours. The dried samples were digested using concentrated HNO_3_ (9 mL) and H_2_O_2_ (1 mL) at 120°C using commercially available temperature-controlled sand digestion technology. The digested suspension was filtered through Whatman No. 42 filter paper, and the trace elements concentrations in the filtered solution were determined using an inductively coupled plasma mass spectrometer (ICP-MS, 7700x, Agilent, USA). A certified soil and plant material (i.e. NIST 2710a and 1570a) was used to ensure the quality of soil and plant digestion analysis.

### Statistical analysis

All of the determinations were performed in triplicate for each independent soil, amendment, and plant sample. One-way analysis of variance (one-way ANOVA) was used to compare the means of the different treatments. Where significant *p*-values (*p*<0.05) were obtained, the differences between the means were evaluated using Duncan’s test. The relationships among the experimental results were evaluated using Pearson’s correlation analysis. The data were analyzed using the SAS program (SAS 9.2, USA).

## Results and Discussion

### Basic properties of soils and amendments

The basic characteristics and pseudo-total concentration of the soils and amendments were determined (Table 1). The pH at Bonghwa (BH), Gwangmyeong (GM), and Danyang (DY) were 5.9, 7.2, and 8.3, indicating acidic, neutral, and alkali soil, respectively. All types of soil were highly contaminated with arsenic and trace elements to varying degrees; in particular, the highest levels of trace elements exceeded the ‘warning levels’ described in the Soil Environmental Preservation Act (25, 4, 150, 200, and 300 mg kg^-1^ for As, Cd, Cu, Pb, and Zn at Area 1, respectively) [17]. Especially in the case of BH and DY, the soils were seriously co-contaminated with As, Pb, and Zn and the GM was highly co-contaminated with Cu, Pb and Zn. Considering the differences in the contaminant species and the degree of contamination, we evaluated the contamination levels using an integrated pollution index (IPI) (Table 1). The IPI was defined as the mean value of the pollution index (PI) of each trace element [11]. In this study, the PI of each element was calculated as the ratio of the metal concentrations in the soils to the background concentration in Korea [18]. Following Yoon et al. [18], the average natural metal contents were 6.83, 0.287, 15.26, 18.43, and 17.68 mg kg^-1^ for As, Cd, Cu, Pb, and Zn, respectively. The IPI was calculated by adding the ratio of each element, and the indexes were 34.9, 34.8, and 32.3 for BH, GM, and DY, respectively. The indexes demonstrated that although there were differences in each contaminant and at each level, the integrated pollution levels were similar among the three types of soils. The similar IPI were useful for minimizing the effect of the contamination differences and for observing the effects of the soil pH.

**Table 1:** Total trace elements concentrations and chemical characteristics of contaminated soils and soil amendments used in the present study.

		pH	EC^a^	LOI^b^	As^c^	Cd^c^	Cu^c^	Pb^c^	Zn^c^	IPI^d^
Soil	Bonhwa	5.9	1.4	1.4	200.2	6.6	89.0	1879.3	782.3	34.9
	Gwangmyeong	7.2	0.6	1.8	12.4	17.4	331.0	981.6	1971.9	34.8
	Danyang	8.3	0.6	3.4	357.5	11.4	34.6	810.2	1246.4	32.3
Amendments	Steel slag	12.4	9.4	0.3	-^e^	4.4	18.6	10.6	204.2	
	Waste lime	12.9	38.8	12.4	6.8	-	11.5	-	31.9	
	Acid mine drainage sludge	8.4	3.8	20.0	-	30.0	-	6.0	966.0	
	Bone meal	7.3	21.5	44.1	-	-	1.9	-	116.4	
	Pig manure	7.8	9.8	58.5	1.7	2.4	55.6	4.6	209.1	
	Spent coffeegrounds char	7.0	3.0	95.5	-	-	19.4	1.6	9.5	

^a^Electrical conductivity (ds m^-1^);

^b^Loss on ignition (%);

^c^Total trace elements concentrations (mg kg^-1^);

^d^Integrated pollution index;

^e^Not detected.

The six amendment types could be divided into two types: inorganic and organic. Inorganic materials (SS, WL, and AMDS) exhibited higher pH levels than those of the organic materials (BM, PM, and SCGC). In particular, the pH of SS and WL exceeded 12, which was a remarkable increase in soil pH. Although the electrical conductivity (EC) was high in the WL and BM, considering the treated amount of soil (2%), the EC level was not so high as to inhibit plant growth in general [19]. In this study, the amendments were applied to soils only after they were air-dried, thus loss on ignition (LOI) indicated both the water content and organic content. The AMDS, the highly concentrated trace elements sludge from the acid mine drainage treatment plant and the organic materials, BM, PM, and SCGC showed high LOI indexes compared with SS and WL. Though the contents of Cd and Zn in the AMDS were high, the effectiveness of AMDS seems to be considerable because the leaching of Cd and Zn was negligible in the experiment and in previous studies using the same AMDS [20].

### Effects of amendments on the characteristics of soils

The pH of the acidic soil (BH) increased significantly (p<0.05) after the addition of all amendment types. However, the neutral (GM) and alkali (DY) soil pH only increased significantly (p<0.05) in the SS and WL amendments, representing a high pH of over 12 (Table 2). Although notable increases in the EC were observed in the WL amendment in all soil types, the EC values resulting from salt stress (4 ds m^-1^) were not high enough to hinder plant growth [19]. The water holding capacity (WHC) was an indicator of the available water content for plants in the soil. The high WHC of the DY from the high content of organic matter (Table 1) was measured as 44%, whereas those of the BH and GM were both 30% (Table 2). Adding most types of amendments has shown the tendency to increase WHC. Karhu et al. [21] noticed that the input of biochar increased the WHC by 11% at a field scale, and Liu et al. [22] revealed that the use of compost and biochar could increase the soil WHC by increasing soil water retention during the dry summer season. This phenomenon occurs not only from organic amendments; Adriano and Weber [23] reported that the application of fly ash could increase both the WHC and plant available water (PAW) at the field scale. It is generally known that the WHC is mainly affected by soil texture, especially fine sand (0.02–0.2 mm) and organic carbon, and that the contents were positively correlated with the WHC [24]. In this study, the increased organic carbon and fine particles caused by adding amendments might have increased the soil WHC and resulted in suitable soil for agricultural land use.

**Table 2:** Changes in the soil chemical characteristics of Bonghwa, Gwangmeong, and Danyang soils after applications of the soil amendments.

	pH	EC^a^	WHC^b^	M3^c^				
				As	Cd	Cu	Pb	Zn
*Bonghwa*								
Control^d^	5.87 d*	1.42 bc	30 d	13.2 b	0.4 a	8.1 ab	427 a	19 bc
SS	7.79 a	1.17 cd	33 c	12.7 b	0.4 a	6.9 bc	290 c	17 cd
WL	7.93 a	2.43 a	34 abc	12.0 b	0.3 a	7.6 b	345 bc	15 d
AMDS	6.23 c	1.52 b	33 c	7.1 c	0.3 a	5.8 c	292 c	24 a
BM	6.85 b	1.04 d	36 a	16.4 a	0.4 a	9.5 a	418 a	21 ab
PM	6.36 c	1.05 d	35 abc	13.1 b	0.4 a	8.4 ab	418 a	19 bc
SCGC	6.35 c	0.94 d	33 bc	12.5 b	0.4 a	7.7 b	386 ab	16 d
*Gwangmeong*								
Control	7.24 b	0.63 cd	30 b	5.1 b	6.2 b	75.1 a	305 c	404 a
SS	7.86 a	0.76 c	29 b	5.0 b	5.5 c	62.7 b	243 e	334 cd
WL	7.81 a	1.46 a	36 ab	5.2 b	5.7 c	63.2 b	278 d	307 d
AMDS	7.25 b	1.06 b	36 ab	2.1 c	5.0 d	57.9 b	209 f	346 bc
BM	7.36 b	0.73 c	35 ab	7.5 a	6.8 a	60.7 b	314 bc	330 cd
PM	7.32 b	0.63 cd	33 b	5.3 b	6.9 a	76.2 a	335 a	377 ab
SCGC	7.29 b	0.57 d	38 a	5.2 b	6.8 a	71.9 a	329 ab	360 bc
*Danyang*								
Control	8.32 cd	0.63 cd	44 d	14.7 b	6.7 bc	3.0 b	52 a	115 bc
SS	8.75 a	0.76 bc	49 c	7.8 d	6.4 c	2.8 b	52 a	102 d
WL	8.49 b	1.83 a	48 c	9.5 c	6.7 bc	2.9 b	50 a	94 e
AMDS	8.18 d	0.84 b	51 bc	2.2 e	4.8 d	2.2 c	28 b	101 d
BM	8.26 cd	0.84 b	52 b	21.5 a	8.8 a	4.0 a	54 a	128 a
PM	8.39 bc	0.63 cd	53 ab	10.2 c	7.5 bc	3.0 b	51 a	113 c
SCGC	8.29 cd	0.51 d	55 a	10.3 c	8.1 b	3.2 b	51 a	121b

*Different letter indicates significant differences at the 5% level by Duncan’s test

^a^Electrical conductivity (ds m^-1^);

^b^Water holding capacity (%);

^c^Mehlich-3 extractable fraction (mg kg^-1^);

^d^SS steel slag, WL waste lime, AMDS acid mine drainage sludge, BM bone meal, PM pig manure, SCGC spent coffee grounds char.

### Changes in bioavailability of trace elements

The Mehlich-3 solution extraction method was developed to test the plant available nutrients in soil in an earlier study, but many recent studies revealed that the Mehlich-3 solution also reflected the bioavailability of soil trace elements [13–16]. The bioavailability of As significantly decreased from AMDS but significantly increased from BM treatments (p<0.05) in all soil types (Table 2). In oxic-soil conditions, as in agricultural upland soil, arsenate was the main chemical species of As in anion forms (H_2_AsO_4_^-^, HAsO_4_^2-^) and easily adsorbed onto Ca -, Fe -, Al—oxides and clay surface sites in soil solid phase [25]. The AMDS used in this study included the sludge from treatment plants where the main process involved the additions of Ca on acid mine drainage resulting in coagulation and precipitation, and the AMDS also contained a large amount of Fe with oxides [20] that might have caused efficient As stabilization. Furthermore, the SS and WL containing Ca and Fe oxides were also good at stabilizing As in the soil [25]. The BM was the slow-releasing fertilizer based on organic phosphorous (P) and was widely used in agriculture over the long-term [26]. As and P have similar characteristics in soil, which causes competitive adsorption on several adsorption sites, resulting in increases in As mobility when utilizing P treatments in As-contaminated soil [27, 28]. Therefore, the increases in As bioavailability represents the results of increased P concentrations after BM treatment. Arsenate mainly exists in the form of H_2_AsO_4_^-^ in acidic and neutral condition and in the form of HAsO_4_^2-^ in alkali condition. In comparison, HAsO_4_^2-^ showed a high degree of mobility or bioavailability in soil. Therefore the change range of As extractability by various types of amendments was wide in alkali soil (2.2 to 21.5 mg kg^-1^) when compared to acidic soil (7.1 to 16.4 mg kg^-1^) and neutral soil (2.1 to 7.5 mg kg^-1^) (Table 2). These results indicated that this type of amendment strongly influenced As stabilization due to the soil pH.

The ratio of Cd bioavailability to the pseudo-total concentrations (6.1%) was too low to observe a significant reduction in acid soil (BH) (Table 2). For neutral soil, the Cd bioavailability significantly decreased under the inorganic amendments SS, WL, and AMDS. The soil pH significantly increased under the SS and WL amendments resulting in decreases in Cd mobility, causing a consecutive reduction in Cd bioavailability. Compared with the SS and WL, the AMDS did not change the soil pH significantly. Nevertheless, the Cd bioavailability also decreased as in previous explanations of adsorption mechanisms [20]. However, compared with acidic and neutral soils, the pH of alkali soil had high representation of low Cd bioavailability. Consequently, it was difficult to observe the effects of pH increases on the reduction in Cd bioavailability by SS and WL; only the AMDS significantly decreased Cd bioavailability. Despite the high content of Cd in AMDS (30.0 mg kg^-1^), any concerns about Cd leaching from AMDS were not observed. In the case of Cd, these results indicated that both pH increases and adsorption mechanisms were exhibited in acid or neutral soils, but in alkali soils, Cd stabilization was mainly preceded by adsorption.

Cu bioavailability significantly decreased from only AMDS for all types of soils (p<0.05), and we could not find any effect of organic amendments (Table 2). Various amendments based on organic matter were applied to the soil for pollutant adsorption and the amelioration of soil quality. Over time, the organic matter could be easily decomposed by soil microbes to produce low molecular organic molecules acting as chelators that could increase the mobility and availability of trace elements [29, 30]. In particular, the property of Cu has a higher affinity for organic carbon or matter that increased Cu bioavailability, so it was difficult to observe any stabilization effects from organic amendments [3, 4].

The Pb bioavailability was significantly reduced by only three types of inorganic amendments in acidic and neutral soils (p<0.05) (Table 2). The stabilization of Pb was observed under the SS and WL treatments, which resulted from the effects of pH increases causing the reduction of Pb mobility. Without any changes in soil pH under AMDS treatment, Pb bioavailability might decrease due to adsorption mechanism of AMDS, indicating the broad range of the applicability of AMDS on trace element stabilization in soil. Nevertheless, pseudo-total Pb contents are similar in neutral soil and alkali soil; furthermore, there has been a considerable difference shown in the ratio of Pb bioavailability to pseudo-total concentrations (31% and 6.4% for neutral and alkali soil, respectively), indicating that Pb in alkali soil exists in a stable form. Therefore, for the same reason as Cd, the significant stabilization efficiency from various amendments on Pb was not easily observed in alkali soil, except for AMDS.

The ratios of Zn bioavailability to pseudo-total concentrations were 2.4%, 20.5%, and 9.2% for acid, neutral, and alkali soil, respectively, indicating that the difference in the bioavailability ratio was greater than the pseudo-total concentrations. In acid soil, the Zn bioavailability significantly increased under AMDS treatment (p<0.05) (Table 2). There are several possible reasons for this result when accounting for the high content of Zn in AMDS (966.0 mg kg^-1^) and the acidic condition of soil pH (6.23 for AMDS), which resulted in Zn leaching from AMDS into the soil. However, the use of AMDS seems to be moderate considering that the increased degree of bioavailable Zn was not very high and that Zn is an essential microelement for plants. Despite the high content of Zn, 204.2 mg kg^-1^, in SS, the result indirectly supports that Zn exists in a stable form. In neutral and alkali soil where the bioavailable portion was relatively high, most of the amendments could stabilize Zn, except for BM and PM. Like AMDS, BM and PM also contained Zn at 116.4 and 209.1 mg kg^-1^, respectively. However, unlike AMDS, Zn existed in an organic form, which is more loosely coupled within an organic than inorganic form that might lead to the Zn leaching during the aging process. Unlike the case of AMDS in acid soil, the Zn leaching phenomenon was not shown and might be masked by the two possible phenomena: the high pseudo-total concentration and the high Zn bioavailability ratio, which demonstrated that the effect of the adsorption of bioavailable Zn onto AMDS was greater than that of Zn leaching from the AMDS.

In sum, through a chemical assessment using the Mehlich-3 extraction method, As stabilization could be achieved through adsorption mechanisms rather than pH control. In the case of cationic trace elements, stabilization was established after pH increases by amendments in acidic and neutral soil. However, in alkali conditions, the effect of adsorption on trace elements stabilization was much greater than that of pH control. The phenomenon of increased trace element bioavailability was partially observed but did not reach a serious level. Moreover, for successful chemical assessments of stabilization efficiency, not only the absolute quantity of the measured value but also the ratio of available content to total content should be considered enough.

### Plant growth and trace elements uptake

The chemical stabilization of trace elements in soil using various amendments was observed through previous chemical assessments. In terms of agriculture, a phytotoxicity test was conducted to make certain that, like former reductions in the bioavailability of trace elements and the input of organic-based amendments, the application of various amendments really worked on safer food crop production using bok choy (*Brassica campestris* L. ssp. chinensis Jusl.), which is a common leaf and stem vegetable in Asia, including Korea.

Phytotoxicity has been defined as any adverse effect on plants resulting from specific substances or growth conditions. After seed germination in the soil, the early growth stage of plants has been deemed important for the continuous growth of plants, and relevant experiments have been studied on various research fields [20, 31]. Bok choy was cultivated on amended soil over 2 weeks, and the shoots or edible parts of bok choy were analyzed (Table 3). Despite a similar pollution index among the three types of soils and the same cultivation period and conditions, the fresh weight and growth length of bok choy were relatively high in the acidic soil, BH. This result might be attributed to the relatively low bioavailability ratio and the acidic conditions that are an advantage for nutrient availability in terms of the nutrient uptake between the soil and plant [32, 33]. However, for each soil, the changes in the fresh weight and growth length from each type of amendments were not as significant as those of the bioavailability using the Mehlich-3 solution.

**Table 3:** Results of the phytotoxicity test conducted by short-term cultivation using bok choy (*Brassica campestris* L. ssp. chinensis Jusl.).

	FW^a^	SE^b^	As^c^	Cd	Cu	Pb	Zn
*Bonghwa*							
Control^d^	99 a*	7.2 ab	0.47 b	0.35 a	2.82 ab	3.20 a	14.2 a
SS	94 a	8.4 a	0.57 ab	0.07 d	2.64 ab	3.84 a	7.2 bc
WL	98 a	8.5 a	0.70 ab	0.09 d	1.78 c	3.57 a	4.3 c
AMDS	95 a	7.9 a	0.55 ab	0.15 c	1.61 c	3.22 a	7.4 bc
BM	46 c	6.1 b	1.06 a	0.14 c	2.29 bc	4.93 a	9.6 b
PM	78 ab	6.7 ab	0.72 ab	0.28 b	2.11 c	4.00 a	9.6 b
SCGC	61 bc	5.4 b	0.66 ab	0.31 ab	3.55 a	3.66 a	9.4 b
*Gwangmeong*							
Control	53 bc	5.5 b	0.10 a	0.98 bc	3.92 a	1.83 a	22.5 ab
SS	44 c	5.5 b	0.10 a	0.90 bc	3.18 a	2.30 a	18.7 ab
WL	70 ab	6.9 ab	0.10 a	0.95 bc	3.83 a	1.75 a	15.2 b
AMDS	53 bc	5.9 ab	0.08 a	0.66 c	3.67 a	1.85 a	17.6 ab
BM	85 a	7.5 a	0.09 a	1.15 b	3.16 a	1.65 a	17.2 ab
PM	59 bc	6.1 ab	0.11 a	1.56 a	4.07 a	2.41 a	25.1 a
SCGC	57 bc	5.5 b	0.11 a	1.19 b	3.11 a	1.68 a	15.3 b
*Danyang*							
Control	44 ab	5.3 ab	1.14 a	2.24 a	3.61 ab	0.67 a	23.8 a
SS	38 bc	4.7 ab	1.13 a	0.62 c	4.25 a	0.63 a	13.0 b
WL	53 a	6.1 a	0.75 b	1.37 abc	6.72 a	0.67 a	16.7 ab
AMDS	36 bc	4.4 b	0.91 ab	1.13 bc	4.10 a	0.65 a	19.9 a
BM	40 b	5.0 ab	1.04 ab	0.79 bc	3.75 ab	0.83 a	20.3 a
PM	45 ab	5.1 ab	0.78 b	1.48 abc	3.19 b	0.49 a	19.6 a
SCGC	29 c	4.9 ab	1.04 ab	2.19 ab	3.73 ab	0.68 a	24.4 a

*Different letter indicates significant differences at the 5% level by Duncan’s test

^a^Fresh weight of bok choy shoot (mg seedling^-1^);

^b^Shoot elongation of bok choy(cm seedling^-1^);

^c^Trace elements concentrations in bok choy shoot (mg kg^-1^) based on fresh weight;

^d^SS steel slag, WL waste lime, AMDS acid mine drainage sludge, BM bone meal, PM pig manure, SCGC spent coffee grounds char.

The trace element concentrations in the bok choy shoots were at a range of 0.08 to 1.14, 0.07–2.24, 1.61–6.72, 0.49–4.93, and 4.3–25.1 mg kg^-1^ for As, Cd, Cu, Pb, and Zn, respectively (Table 3). The uptake and accumulation of trace elements were relative high in Cu and Zn because they are essential micronutrients for plant physical development [33]. The As concentration in acid soil by bok choy significantly increased only from BM (p<0.05). This result was attributed to the significant changes in the bioavailability of As in response to the BM treatment, which increased the As mobility by competitive adsorption [29, 30]. The Cd and Zn concentrations in the shoots of bok choy were significantly lower compared to the control in acid and alkali soils, with the exception of a few treatments. The reduction in the uptake amount was high in the SS and WL treatments where the pH increased the most. The changes and tendency of Cu uptake were also similar to the former results. However, for Pb, no significant change occurred in the uptake. Hettiarachchi and Pierzynski [34] found that the solubility of Pb in the soil environment was low and the rate of Pb translocation from roots to shoots was also slow. Because of these behavioral properties of Pb, it was hard to identify the changes in uptake concentrations from amendment treatments. Considering that this result and tendency also appeared in the case of acid soil, it seemed that the achievement of remediation would be easy to secure for the purpose of safer food crops.

Table 4 presents the relationships among the results in this study. However, there were significant differences in the types of pollutants and those of the concentrations among the three soils. Therefore, we used a ratio (%) of the bioavailability to the pseudo-total concentration, rather than the absolute quantity (mg kg^-1^) of trace element bioavailability. The results indicated that the uptake of Cd, Pb, and Zn by bok choy significantly increased with an increasing ratio of bioavailability to pseudo-total concentrations (p<0.05 for Pb, p<0.001 for Cd and Zn). The correlation coefficient was lowest in Pb due to the low mobility between soil-plant [34, 35]. Cu is an essential micronutrient, and its behavior has been highly dependent on not only the pH but also the dissolved organic carbon, resulting in a significant correlation between the results of chemical assessment and biological assessment [3, 20]. However, As in the shoots was significantly negatively associated with the ratio of the bioavailable fraction (p<0.001) (Table 4). This negative relationship could be accomplished by the greater difference in the pseudo-total concentration than in the bioavailability among the three types of soils. When calculating the ratio, the As bioavailability (the numerator) in alkali soil (14.7 mg kg^-1^) was almost 3-fold greater than that in neutral soil (5.1 mg kg^-1^) (Table 2). However, the pseudo-total concentration in alkali soil (the denominator) (357.5 mg kg^-1^) was 30-fold greater than that in neutral soil (12.4 mg kg^-1^) (Table 1). Therefore, the negative correlation coefficient resulted from a relative undervaluation of the effects of As in alkali soil due to a 10-fold difference that was only caused by the differences in the pseudo-total concentration.

**Table 4:** Correlation coefficients (*r*) among the results of the present study.

	As^a^	Cd	Cu	Pb	Zn
P_As_^b^	-0.8383***				
P_Cd_		0.9508***			
P_cu_			0.1373		
P_Pb_				0.4240*	
P_Zn_					0.7628***

*Represent significant at *P* < 0.05, and

*** represent significant at *P* < 0.001, respectively, according to *Pearson* correlation analysis

^a^Concentrations of each trace elements in bok choy shoot;

^b^Percentages (%) of bioavailable fraction (Mehlich-3 extractable concentrations / total concentrations × 100).

From these results, we surmised that the tendency of trace element translocation from soil to plant was well demonstrated by the portion of the bioavailable pool of trace elements, except in cases where the total concentration was far different among soil samples.

## Conclusion

This study evaluated the effects of various amendments on trace element stabilization and phytotoxicity in different soil pH levels contaminated by trace elements with similar pollution levels using both chemical and biological assessments. Inorganic amendments containing Fe or Ca-based adsorption sites were useful for stabilizing As in all soil pH types. For cationic trace elements, the effectiveness of stabilization by several amendments was revealed in acidic or neutral soils. The AMDS exhibited a great ability to stabilize most trace elements. In a phytotoxicity test, the uptake of trace elements by bok choy shoots was affected by the portion of the bioavailable fraction. Therefore, when using a stabilization method for soil remediation, one should be concerned not only with the types of amendments but also with the bioavailable portion of trace elements. However, it is difficult to determine the clear effects of organic amendments due to the short-term cultivation conditions and the relative comparison with inorganic amendments; future research requires more numerous and long-term cultivation studies.
